# Challenges Involved in Caring for a Loved One Who is Living with Dementia and the Potential Appropriateness of an Innovative Gaming Activity for Social and Emotional Wellness: Exploring Focus Group Results from Caregivers of Clients Involved in a Group Digital Gaming Intervention

**DOI:** 10.1002/alz.092499

**Published:** 2025-01-09

**Authors:** Michelle D Hand, Megumi Inoue, Naoru Koizumi, Emma Booker, Sarah Nosrat, Samreen Mehak, Eyesha Shaikh

**Affiliations:** ^1^ George Mason University, Fairfax, VA USA

## Abstract

**Background:**

Slowing the progression of dementia and maintaining mental and social wellness are important for people living with dementia (PWD) and their caregivers. Social engagement can help with delaying the onset of dementia among people living with mild cognitive impairment. Maintaining meaningful social engagement through activities, such as gameplay, can promote a sense of belonging and identity while maintaining mental functioning. Physical activities can also improve strength and cognitive outcomes in PWD. Nursing homes, assisted living facilities, and adult day health centers provide various types of activities that encourage social, physical and mental wellness for PWD (e.g., through gameplay). A group gaming system, Obie Technology, was developed to promote these activities, to facilitate movement, cognitive exercises, and social interaction. This study explored the potential appropriateness of a group gaming intervention on cognitive function, mood, and behavior in PWD. The qualitative focus group results with caregivers will be presented.

**Method:**

Included were caregivers of clients in an adult day health center who were selected to participate in a group digital gaming intervention. All clients that were cared for by the participants were living with mild‐ or moderate‐stage impairment. Three semi‐structured focus groups were conducted, to discuss what it is like to care for someone living with mild‐ or moderate‐stage dementia, client mood, and participant questions surrounding the gaming intervention.

**Results:**

The three focus groups lasted approximately one hour each, and were conducted with a total of 21 participants, who were caring for PWD selected for the group gaming intervention. The results illuminate caregiver perceptions surrounding their challenges in providing care to PWD and the appropriateness of the gaming intervention for PWD.

**Conclusion:**

Challenges were discussed surrounding changes in relationships with loved ones, who were primarily spouses or parents of the participants. Participants also discussed challenges surrounding anger and aggression expressed by clients toward them as caregivers. Concerns were expressed about the possibility of the digital gaming intervention resulting in falls among the clients as well, adding to these challenges. These results will be further discussed, along with implications for research, practice, and policy centered around PWD and their caregivers.

